# Long-Term Trends of Liver Cancer Incidence and Mortality in China 1990–2017: A Joinpoint and Age–Period–Cohort Analysis

**DOI:** 10.3390/ijerph16162878

**Published:** 2019-08-12

**Authors:** Fang Wang, Sumaira Mubarik, Yu Zhang, Lu Wang, Yafeng Wang, Chuanhua Yu, Hao Li

**Affiliations:** 1 Department of Epidemiology and Biostatistics, School of Health Sciences, Wuhan University, Wuhan 430071, China; 2 School of Public Health, Tongji Medical College, Huazhong University of Science and Technology, Wuhan 430030, China; 3 Medical College of Hubei University of Arts and Science, Xiangyang 441053, China; 4 Global Health Institute, Wuhan University, Wuhan 430072, China

**Keywords:** liver cancer, incidence, mortality, Joinpoint regression analysis, age–period–cohort model, trends

## Abstract

Liver cancer (LC) is one of the most common causes of cancer-related deaths: this study aims to present the long-term trends and age–period–cohort effects of the incidence of and mortality from LC in China during 1990–2017. Incidence and mortality data were obtained from the Global Burden of Disease Study 2017. We determined trends in the age-standardized incidence rate (ASIR) and mortality rate (ASMR) using Joinpoint regression. An age–period–cohort (APC) analysis was performed to describe the long-term trends with intrinsic estimator methods. The ASMR decreased markedly before 2013 and increased thereafter, with overall average annual percent change (AAPC) values of −0.5% (95% confidence interval (CI): −0.6%, −0.3%) for men and −1.3% (−1.6%, −1.0%) for women during 1990–2017. The ASIR significantly increased by 0.2% (0.1%, 0.3%) in men and decreased by 1.1% (−1.2%, −1.0%) in women from 1990 to 2017. The risks of LC incidence and mortality increased with age in both genders. The period effect risk ratios (RRs) of incidence and mortality displayed similar monotonic increasing trends in men and remained stable in women. The cohort effect showed an overall downward trend and almost overlapping incidence and mortality in both genders, and later birth cohorts experienced lower RRs than previous birth cohorts. Older age, recent period, and birth before 1923 were associated with a higher risk of liver cancer incidence and mortality. The net age and period effects showed an increasing trend, while the cohort effects presented a decreasing trend in incidence and mortality risk. As China’s population aging worsens and with the popularization of unhealthy lifestyles, the burden caused by liver cancer will remain a huge challenge in China’s future.

## 1. Introduction

As countries develop, the burden of noncommunicable diseases (NCDs), including cardiovascular diseases and cancers, increases. Generally, cancer incidence and mortality are rising continuously, now occupying second place in the rankings of global deaths, years of life lost (YLLs), and disability-adjusted life years (DALYs) [1,2].

Liver cancer (LC) is the sixth most commonly diagnosed cancer and the fourth-leading cause of cancer death worldwide, causing about 0.84 million new cases and 0.78 million deaths in 2018 [3,4]. Among all diseases, the rank of LC mortality has risen from 30th in 1990 to 19th in 2017 (annual change in death: 0.91% per 100,000), accounting for 1.46% of all deaths per 100,000 in 2017 [2]. The “Global Burden of Disease (GBD) 2015” summarized the burden and underlying etiologies of primary LC at the regional and national level and found that the highest burden of LC incident cases, deaths, and DALYs were observed in East Asia. High-income Asia Pacific and Western Europe respectively ranked second and third for LC incident cases. Southeast Asia experienced the fourth highest number of incident LC cases, but ranked second for LC deaths and DALYs [4]. In China, from 1990 to 2017, the LC incidence rates increased from 21.56 to 36.52 per 100,000, while deaths increased by 44.46% (from 20.49 to 29.6) [5]. According to the GBD 2017, 0.52 million cases and 0.42 million deaths occurred in China, ranking it third for incidence and sixth for mortality. Some studies have showed that LC remains an important public health problem: A study from Australia showed that incidence and mortality associated with LC increased substantially from 1982 to 2015 [6,7,8,9]. Previous studies of most Eastern or Southeastern Asian populations have reported that although the incidence rate of LC is predicted to decline, the burden of LC will continue to increase with the growth and aging of the population [10,11].

China accounts for a significant proportion of cancer deaths due to the rise in cancer incidence and mortality. In China, the burden of LC has become a severe public problem that has accompanied rapid socioeconomic development, changes in lifestyle, and the aggravation of population aging. The burdens of LC were estimated at 0.42 million excess deaths, with 11 million years of life lost (YLLs) and 0.13 million years lived with disability (YLDs) according to the GBD 2017. This has a massive effect on the global variation trend of LC and its burden, which deserve more profound studies. On the other hand, the change in LC incidence and mortality might reflect different etiologies and risk factors in different periods. From a pooled population-based cancer survival study, the age-standardized five-year relative survival rate of LC patients was 12.1% in China (12.2% for males and 13.1% for females) [12]. The survival rate was generally low and flat, which is indeed a significant problem, not only in China, but globally [13]. The prognosis for LC patients is inferior, and prevention, early detection, and treatment are the primary means to reduce mortality [14]. This premise requires an in-depth analysis of the trends of LC incidence and death during the past decades in China, exploring the underlying causes. Therefore, we conducted a study on the prevalence trend of LC.

As far as we know, current studies of LC incidence or mortality have been mainly limited to specific regions and populations or have focused on the short-term trends of a particular indicator of mortality or morbidity [15,16,17,18], and most research globally has used data from national cancer surveillance agencies, such as the Chinese National Cancer Center, the U.S. Census Bureau, or Statistics South Africa, which has some limitations for global comparison [19,20]. However, some studies have only analyzed crude trends, and besides, few investigations have studied the simultaneous incidence and mortality trend of liver cancer in China by using age–period–cohort (APC) analysis, which can separate age, period, and cohort effects [21]. We herein present results from the GBD 2017 study and provide an assessment of the up-to-date trends of LC incidence and mortality in China from 1990 to 2017 by using an age–period–cohort (APC) and Joinpoint analysis.

## 2. Materials and Methods

### 2.1. Data Sources

National liver cancer incidence and mortality (1990–2017) were extracted from the GBD 2017, which was provided by the Institute for Health Metrics and Evaluation (IHME, http://www.healthdata.org/). IHME is an independent global health research center at the University of Washington. The GBD estimates the metrics of incidence, prevalence, death, YLLs, YLDs, and DALYs of each disease and injuries, which are reported by year, location, age group, and sex. The purpose of the GBD study is to establish comprehensive and comparable global health metrics. In China, original data were collected from the literature; Vital Registration, Chinese Vital Statistics (deaths); the Ministry of Health, Cancer Registry; and the World Health Organization (WHO) Mortality Database; among other sources. The registry system, data collection procedure, and estimation processes were previously described in detail [1]. Therefore, as it was strictly checked and verified at all levels of departments, the quality of data was highly reliable.

### 2.2. Incidence and Mortality Data of Liver Cancer

The types of LC in this study included liver cancer due to hepatitis B, hepatitis C, alcohol use, nonalcoholic steatohepatitis (NASH), and other causes. LC was defined according to the Ninth Revision of the International Classification of Diseases (ICD-9) (code 155) and ICD-10 (coded as C22). Age-standardized incidence rate (ASIR) and mortality rate (ASMR) in both genders were based on the GBD 2017 global age (standard population). To estimate the age, period, and cohort effects, the ASIR and ASMR data of LC were extracted and categorized into consecutive 5-year periods from 1992 to 2017 and 15 5-year age groups from 20 to 24 years to 90 to 94 years.

### 2.3. Statistical Analysis

#### 2.3.1. Age–Period–Cohort Analysis

The APC model described the trends of LC incidence and mortality according to age, period, and cohort every five years. Collinearity is a general problem in the application of an APC model because of the cohort = period–age relationship. The method based on the intrinsic estimation (IE) algorithm, which has been confirmed for its parameter estimability, nonbias, validity, and asymptoticity, was applied in this study [22]. The model was expressed as: *Y* = log(*M*) = *μ + αage_i_ + βperiod_j_ + γcohort_k_ + ε*. *M* is defined as the incidence or mortality rate; α, β, and γ refer to the coefficients of three dimensions (α refers to the age effect, that is, the risk of death or morbidity in a particular age group; β is the period effect, which is the risk of mortality or morbidity in a given period for people; and γ is the cohort effect, the risk of death or morbidity for all people in the same birth cohort); and μ and ε are defined as the intercept and random error.

The APC model was performed through Stata 15.0 software (Stata Corp, College Station, TX, USA). The degree of model fitting was evaluated through deviance, Akaike’s information criterion (AIC), and the Bayesian information criterion (BIC). Standard error (SE) coefficients and risk ratios were calculated.

#### 2.3.2. Joinpoint Regression Analysis

Joinpoint regression analysis was used to estimate the temporal trends in the ASIR and ASMR of LC. We identified years in which the pattern changed significantly and estimated the annual percentage change (APC), average annual percent change (AAPC), and 95% confidence interval (CI) for each trend segment identified by the model. A maximum number of four points was allowed, ensuring the results were credible. Joinpoint analysis was performed using Joinpoint regression program version 4.6.0.0 (April 2018) (Information Management Services, Inc., Calverton, MD, USA) from the Statistical Research and Applications Branch of the Surveillance Research Program of the U.S. National Cancer Institute.

## 3. Results

### 3.1. Descriptive Analysis of Incidence and Mortality Trends

**Figure 1:**
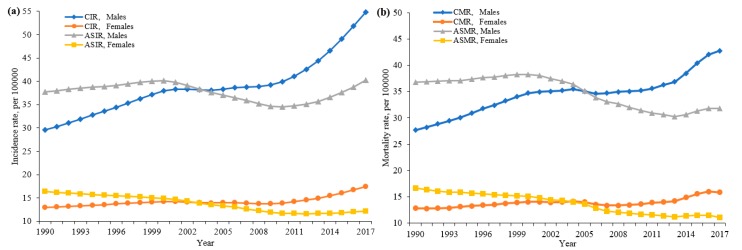
Trends of the CIR, ASIR (**a**), CMR, and ASMR (**b**) of LC by gender, 1990–2017, China (LC: liver cancer; CIR: crude incidence; CMR: crude mortality; ASIR: age-standardized incidence rate; ASMR: age-standardized mortality rate).

Figure 1 shows crude and age-standardized incidence and mortality of LC in the Chinese population from 1990 to 2017. The ASMR decreased from 26.72 in 1990 to 21.30 in 2017 per 100,000 persons, and the ASIR declined slowly from 27.16 to 26.04 per 100,000 during the same period, with a tiny fluctuation. The ASMR decreased markedly before 2013 and increased thereafter, with overall average annual percent change (AAPC) values of −0.5% (95% CI: −0.6%, −0.3%) for men and −1.3% (−1.6%, −1.0%) for women during 1990–2017. The ASIR significantly increased by 0.2% (0.1%, 0.3%) in men and decreased by 1.1% (−1.2%, −1.0%) in women from 1990 to 2017.

Table 1 presents the annual percentage change (APC) and average annual percent change (AAPC) of LC incidence and mortality by gender from 1990 to 2017. Joinpoint regression results show that the ASMR decreased from 2000 to 2013 and then rose until 2017. This trend can be seen in both men and women, with overall AAPC values of −0.5% (−0.6%, −0.3%) for men and −1.3% (−1.6%, −1.0%) for women.

In males, the ASIR increased slowly by 0.7% per year from 1990 to 2000, then decreased by −1.7%, and then increased from 2010 to 2017. The AAPC during the whole period was 0.2% (0.1%, 0.3%). In females, the ASIR decreased from 1990 to 2012, with different APC values, and then increased by 0.9% from 2012 to 2017 (Table 1).

For age-specific rates, mortality decreased in all age groups except for those aged 75 years and older in total, those aged 60–69 years in men, and those aged 90–94 years in women, with a slightly increasing trend. The incidence declined in most age groups (see Appendix Table A1).

### 3.2. Age–Period–Cohort Analysis

#### 3.2.1. Age Effect

**Figure 2:**
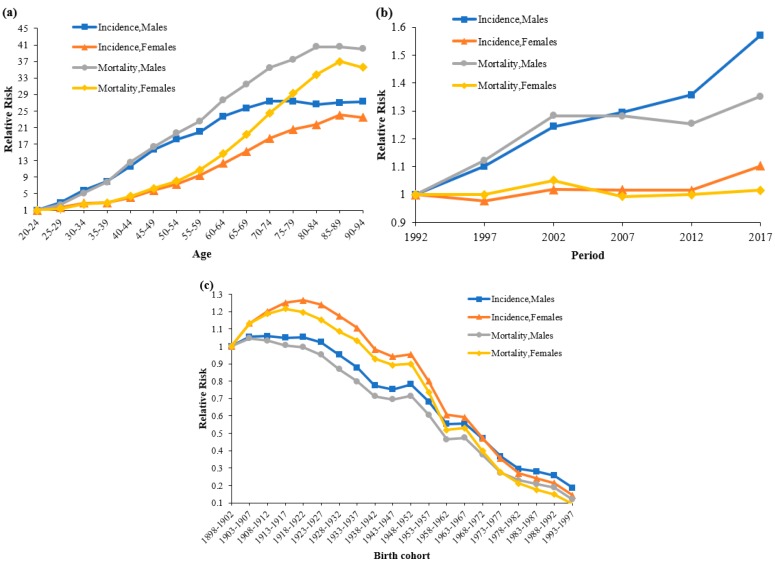
Incidence and mortality relative risks of liver cancer due to (**a**) age, (**b**) period, and (**c**) cohort effects, stratified by gender.

Figure 2a shows the estimated risk ratios (RRs) of incidence and mortality stratified by gender. Generally speaking, the risk of LC incidence substantially increased with age for both genders, except for the 80–84 age group in men, with a slight decrease for the 75–79 age group (Table 2). The risk of LC incidence in the 90–94 age group was about 27.28 and 23.44 times higher than that of the 20–24 age group for men and women, respectively. When it came to the risk of LC mortality, it was about 40.06 and 35.61 times higher, and the age effect pattern of RRs was similar between mortality and incidence.

#### 3.2.2. Period Effect

The period effect RRs displayed similar monotonic increasing trends for incidence and mortality in men (Figure 2b and Table 2). The RR mortality declined slightly in men between 2007 and 2012. Compared to the period group in 1992, the RRs of incidence and death in the period group in 2017 in men were 1.57 and 1.35, respectively. For women, the period effects of incidence and mortality rates were relatively stable.

#### 3.2.3. Cohort Effect

Figure 2c shows the cohort rate ratios of LC incidence and mortality in each cohort. The curves show an overall downward trend and almost an overlap in incidence and mortality for both genders. Later birth cohorts experienced lower RRs than previous birth cohorts, except for some individual cohorts. In terms of incidence, the risk increased before 1922, then declined after 1923 for both genders, except for the 1952 birth cohort in women (Table 2).

In men, the RRs of mortality showed an overall downward trend, except for the 1948–1952 birth cohort. In women, the RRs of mortality showed an increasing trend in the 1903–1917 and 1963–1967 birth cohorts, and then kept decreasing in other cohorts. Although the peak risks of incidence and mortality varied between the different groups, the curves remained subsequently converged afterwards for all groups.

## 4. Discussion

Our findings revealed that, compared to 1990, the ASIR and ASMR of LC in 2017 declined in both genders, and the decline rate of death was higher than that of incidence, but was still at a high level. In addition, both the ASIR and ASMR showed an upward trend in recent years after falling obviously to the lowest level. Hence, it is still necessary to analyze the patterns of LC incidence and the mortality rate and to explore the etiology, natural history, and disparities behind these trend changes.

Due to the interaction between age, period, and cohort effects, we used an age–period–cohort regression model and an intrinsic estimator algorithm to analyze and estimate the influence of age, period, and cohort on the time trends of LC onset and death, and we further explored the causes and influencing factors behind the phenomenon. The results show that the incidence and mortality rates increased with age and period, but decreased with birth cohort. Older age, recent period, and birth before 1923 were associated with a higher risk of LC incidence and mortality.

### 4.1. Age Effects

Age effects were an essential factor affecting LC incidence and death, suggesting the influence of demographic changes on LC morbidity and mortality. As mentioned earlier, the age-related effects of LC incidence and mortality generally increased rapidly in both sexes with age and presented a slight declining trend in women after 89 years of age. That is, increasing age, incidence, and death risk from LC gradually increased, which is consistent with a study conducted by Dal et al. in Italy, and similar patterns can be found in some other studies both in China and globally [17,23,24,25]. For males, the effect value of LC incidence and mortality increased to be positive after the age of 45, and females were 55 years old or older. That is to say, age became a contributing factor to death and morbidity from LC in men after 45 years of age and in women after 55 years of age.

With increasing age, the elderly are more vulnerable to diseases, injuries, air pollution, and other risk factors. On the other hand, the cumulative exposure of the elderly population to risk factors for LC is also increasing, which also has an impact on LC incidence and death. China now has an aging society: from 1992 to 2017, the proportion of the elderly population aged over 65 years in China rose from 6.2% to 11.4%. According to the United Nations (2013), the population aged 65 and over globally will increase to 16% in the next 30 years (2020 to 2050), and by 2050, there will be 400 million Chinese citizens aged above 65, 150 million of whom will be more than 80 [26]. It is conceivable that the increase in aging levels will increase the number of China’s high-risk groups in the future, and the burden of LC will continue to grow.

### 4.2. Period Effects

From the period effects, it can be seen that from 1992 to 2017, the risk of morbidity and mortality in men increased steadily, while the RRs of women remained relatively stable, with no significant fluctuations. The risks of LC incidence and death increased to 1.57 and 1.35 in males from 1992 to 2017. It follows that, in men over the age of 20, the period effects of LC incidence risk increased by 57% over 25 years, and the risk of death increased by 35%. Among women, the increased risks of LC incidence and mortality did not significantly change.

The period effects were affected by a series of complicated historical events and environmental changes, including economic levels, environmental factors, lifestyle behaviors in different periods, disease screening, detection levels, and other factors. From the perspectives of social development (with the reform and opening up of China) and economic development coupled with the implantation of primary national medical and health reform measures and the continuous improvement of medical technology, people’s living conditions have been greatly improved. Advances in serological detection techniques and imaging technologies have improved the initial treatment rate of LC. Progress in basic research, such as the in-depth study of epidemiology and pathogenesis, has provided multiple therapeutic strategies for the clinical prevention and treatment of LC. The diversification of surgical treatment methods and the improvement of technical levels such as liver transplantation have improved the general treatment levels of LC and have further reduced mortality. From these perspectives, the incidence and mortality of LC were supposed to decrease with time, but we found that the results were inconsistent with expectations.

Overall, we consider that the discrepancy between mortality rates and expectations may have been due to increased risks from unhealthy lifestyle behaviors and environmental degradation since the 1990s. Since the end of the last century, Chinese diets have changed vastly, and people have tended to eat more unhealthy food, such as processed meat, fast food, and sugar-sweetened beverages. Moreover, with a high prevalence of smoking, drinking, and lack of exercise [27,28], these changes have also led to the loss of health, high incidences of illness, and early death. Furthermore, disease patterns have undergone epidemiological transitions, with a decline in deaths from infectious diseases and malnutrition, and there has been a rise in the percentage of the disease burden due to noncommunicable diseases (NCDs), such as neoplasms and injuries. Men are more susceptible to adverse lifestyles such as smoking and drinking, and thus they showed an increased risk of death, while women showed a more stable period effect.

On the other hand, in terms of incidence risk, due to the advancement of serological detection and imaging techniques in LC diagnosis, in addition to the impact of environmental degradation and unhealthy lifestyles, the early diagnosis rate of LC has also improved [29]. For example, since the 1990s, serum alpha-fetoprotein (AFP) and ultrasound have been used in LC early diagnosis. Especially in men with chronic hepatitis B or cirrhosis who are over 40 years old (a high-risk group), regular examinations have improved the diagnosis rate of subclinical stages and early LC, which is also manifested as an increased risk of LC. In addition, we found that men and women had different period effects (monotonic increase in men and stable in women), as men were more likely to be affected by modern lifestyle risk factors such as smoking, alcoholism, and other unhealthy behaviors, showing a higher risk. Women, on the other hand, were more likely to be affected by the changing times. Compared to the past, women’s risks have not fluctuated greatly in recent years and have been relatively stable [30].

### 4.3. Cohort Effects

Cohort effects reflect changes in early living environments and assume that people in the same birth cohort have an equal chance of being exposed to disease risk factors. Exposure to certain adverse environmental factors early in life may adversely affect later life. The birth cohort effect obtained by the APC model was the net effect related to birth date after deducting the influence of age and period effects. The birth cohort effects showed that the risk of LC incidence and mortality in the population born since 1898 showed a decreasing trend on the whole, accompanied by a slight fluctuation in individual birth: this was inconsistent with research by Jiang, X., in Canada, but Sun, Y., et al. also found that cohort effects all showed an overall downward trend and almost overlapped for both genders in China [17,25]. Compared to the group born from 1898 to 1902, the LC incidence and death risk in the population born in 1993–1997 decreased by more than 80%.

**Figure A1:**
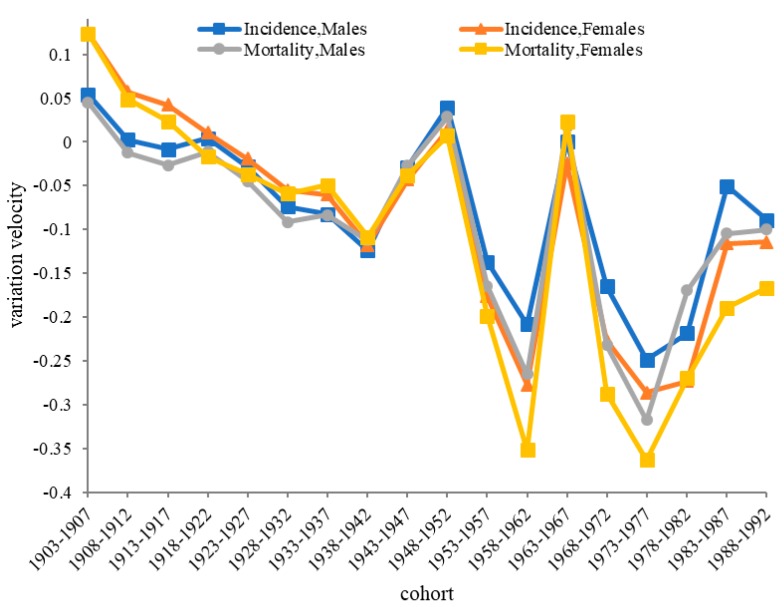
Variation velocity of cohort effects on LC incidence and mortality during 1990–2017.

On the whole, there were three periods of deterioration in the risk of LC events and death, with a rate that slowed down (see Appendix Figure A1). The first stage was 1938–1942 to 1948–1952, which is considered to be related to the Japanese invasion of China and the War of Resistance against Japanese Aggression. The “Lugouqiao incident”, which was outrageously launched by Japan in 1937, raised the curtain on a war of aggression against China, putting China into a long-term period of war. The living environment deteriorated, medical and health services were severely damaged, people were displaced, and their lives and health could not be guaranteed.

The second stage was 1958–1962 to 1963–1967: A series of social and economic system changes took place in China during this period, such as the Great Leap Forward and the People’s Commune Movement. National productivity was severely damaged, the economic level was dysfunctional, and the economy was sluggish. Coupled with a three-year famine from 1959 to 1961, food shortages were particularly acute, and various health risk factors significantly increased [31]. The third stage was 1973–1977 to 1983–1987, which may have been due to some drawbacks brought about by China’s various exploratory reforms in healthcare starting in 1978 [32,33], such as a low efficiency in health input, an increasing contradiction between the supply of and demand for medical resources, and the difficulty and high cost of medical services.

It is worth noting, however, that the overall risk of the birth cohorts was reduced. During the three accelerated decline periods, China established and developed an urban and rural medical security system. At the beginning of the founding of the People’s Republic of China (PRC), China followed the example of the Soviet Union model and established an employee medical security system, including a public medical system in 1952 and a labor insurance medical system in 1953 [34,35]. The medical insurance system for urban workers successively improved, and a rural cooperative medical security system was established. After the second domestic revolutionary war, China launched a patriotic public health campaign, including garbage removal, toilet construction, water well improvement, diverting channels, and sewage pond filling. It dramatically improved urban and rural housing, food, water, healthy environments, and public sanitation, reducing the risk of LC [36]. With the continuous development of the social economy, the popularization of health education knowledge became more and more widespread, people’s health awareness improved, and new nutritional status was enjoyable, which also partly explains the risk reduction in the recent birth cohort.

In terms of gender differences, we found that in the early birth cohort, the risk of morbidity and mortality in women was higher than in men. The risk of female morbidity began to be lower than that of males after the 1963–1967 birth cohort, and the risk of death in the birth cohort after 1968–1972 began to be lower in women than in males. The ASIR and ASMR of men were higher than those of women (Figure 1), which may have been due to the higher prevalence of risk factors and differences in sex steroid hormones, immune responses, or epigenetics between men and women [11,37]. The underlying cause may have been that the main risk factors for LC are different at different birth periods. Women in the early birth cohort had low social status, poor nutrition, and were more susceptible to the social environment, while men born later were more likely to be exposed to smoking, drinking, and other risk factors that lead to a higher level of risk [38]. Based on previous studies reported in China, the unfavorable trend in the group of men born later was related to social disintegration, as they were more likely to be exposed to risk factors such as alcohol and drug abuse, tobacco use, unemployment, and income inequality: the complex interactions between these factors may have led to an increase in LC risk in China [27,39,40,41,42,43].

Epidemiological and experimental studies have shown that the hepatitis B virus (HBV), hepatitis C virus (HCV) infection, aflatoxin exposure, water pollution, unhealthy diet habits, alcohol, cirrhosis, sex hormones, nitrosamines, blood transfusions, and other substances are associated with liver cancer [44,45,46]. In China, HBV infection is recognized as a significant carcinogenic factor, and aflatoxin and water pollution are essential cancer-promoting factors [47].

Since the 1970s, China has proposed the seven-character policy of “manage water and grain, prevent hepatitis” to prevent LC. In 1992, it launched the immunization of newborns and children with the hepatitis B vaccine nationwide, and the incidence of liver cancer caused by factors such as hepatitis B, aflatoxin, and water pollution decreased [48,49]. According to a nationally representative serologic survey, China’s hepatitis B virus surface antigen prevalence declined by 52% from 1992 to 2014 [50]. However, as mentioned above, since modern times, with epidemiological disease transitions, the main risk factors for LC have also changed. Unhealthy lifestyle behaviors and an increase in the proportion of metabolic diseases have led to a more severe risk of LC incidence and death.

Some limitations should be noted in interpreting our findings. First, this study was based on the most recently updated data from the GBD 2017, which used the incidence data from cancer registries as well as mortality data from vital registration systems or verbal autopsies to produce disease burden estimates: diagnostic accuracy for cause of death data and ascertainment bias in cancer registries remain limitations, which leads to a problem in data accuracy. Second, our research was not individually based, so we did not investigate which factors caused changes in liver cancer events and therefore could not make causal inferences. Despite the limitations, our study is still a meaningful national study that investigated the trends and changes in LC incidence and mortality rates.

## 5. Conclusions

In summary, the birth cohort effect of liver cancer incidence and mortality decreased, whereas the incidence and mortality increased with period and age effects. Age was the most influential factor on liver cancer incidence and mortality. Period effects had a more significant impact on men than on women. The incidence and mortality of liver cancer may continue increasing with age: as China’s population aging worsens and with the popularization of unhealthy lifestyles, the burden caused by liver cancer will remain a huge challenge for China’s future. Primary interventions should be carried out promptly, especially among high-risk groups such as men who smoke or have fatty liver disease.

## Figures and Tables

**Table 1:** Trends in liver cancer incidence and mortality rates by gender in China, 1990–2017.

Segments	Both		Males		Females	
	Year	APC * (95% CI)	Year	APC (95% CI)	Year	APC (95% CI)
ASMR						
trend1	1990–2000	0.0 (−0.1, 0.2)	1990–2000	0.5 * (0.4, 0.6)	1990–2000	−0.9 * (−1.2, −0.6)
trend2	2000–2004	−1.5 * (−2.1, −0.8)	2000–2004	−1.4 * (−2.1, −0.7)	2000–2004	−1.6 * (−2.7, −0.4)
trend3	2004–2007	−3.7 * (−4.6, −2.7)	2004–2007	−3.2 * (−4.1, −2.3)	2004–2007	−4.5 * (−6.2, −2.9)
trend4	2007–2013	−1.4 * (−1.7, −1.2)	2007–2013	−1.5 * (−1.7, −1.3)	2007–2012	−1.6 * (−2.2, −1.1)
trend5	2013–2017	1.3 * (0.8, 1.8)	2013–2017	1.6 * (1.1, 2.0)	2012–2017	0.2(−0.3, 0.8)
AAPC *	1990–2017	−0.8 * (−0.9, −0.6)	1990–2017	−0.5 * (−0.6, −0.3)	1990–2017	−1.3 * (−1.6, −1.0)
ASIR						
trend1	1990–2000	0.2 * (0.1, 0.3)	1990–2000	0.7 * (0.6, 0.7)	1990–2000	−0.9 * (−1, −0.8)
trend2	2000–2010	−1.9 *(−1.9, −1.9)	2000–2009	−1.7 * (−1.7, −1.6)	2000–2006	−2.2(−2.4, −2.1)
trend3	2010–2013	0.9 * (0.5, 1.4)	2009–2012	0.3(−0.2, 0.8)	2006–2009	−3.1 * (−3.4, −2.7)
trend4	2013–2017	2.5 * (2.2, 2.7)	2012–2015	2.4 * (1.9, 2.9)	2009–2012	−0.8 * (−1.4, −0.3)
trend5	-	-	2015–2017	3.5 * (2.6, 4.5)	2012–2017	0.9 * (0.7, 1.1)
AAPC	1990–2017	−0.2 * (−0.2, −0.1)	1990–2017	0.2 * (0.1, 0.3)	1990–2017	−1.1 * (−1.2, −1.0)

Note: * APC, annual percentage change; AAPC, average annual percent change; CI, confidence interval; ASMR, age-standardized mortality rate; ASIR, age-standardized incidence rate.

**Table 2:** Age–period–cohort (APC) model analysis results of liver cancer incidence and mortality in China, by gender.

Variables	Incidence (Coef, 95% CI)		Mortality (Coef, 95% CI)	
	Males	Females	Males	Females
**Age**				
20–24	−2.59 * (−4.98, −0.21)	−2.03 * (−3.58, −0.48)	−2.73 (−6.43, 0.97)	−2.19 (−4.75, 0.36)
25–29	−1.57 *** (−2.33, −0.8)	−1.53 *** (−2.31, −0.76)	−1.9 ** (−3.29, −0.52)	−1.86 * (−3.31, −0.41)
30–34	−0.83 ** (−1.3, −0.36)	−1.04 *** (−1.54, −0.54)	−1.1 ** (−1.90, −0.30)	−1.29 ** (−2.18, −0.40)
35–39	−0.52 ** (−0.91, −0.13)	−1.00 *** (−1.43, −0.57)	−0.68 * (−1.32, −0.04)	−1.17 ** (−1.9, −0.45)
40–44	−0.14 (−0.47, 0.19)	−0.63 *** (−0.95, −0.32)	−0.2 (−0.74, 0.33)	−0.73 ** (−1.26, −0.19)
45–49	0.16 (−0.12, 0.45)	−0.27 * (−0.52, −0.02)	0.06 (−0.4, 0.51)	−0.36 (−0.79, 0.06)
50–54	0.31 * (0.06, 0.55)	−0.05 (−0.26, 0.15)	0.24 (−0.15, 0.63)	−0.11(−0.45, 0.22)
55–59	0.4 *** (0.19, 0.61)	0.21 * (0.05, 0.37)	0.38 * (0.06, 0.71)	0.18 (−0.08, 0.44)
60–64	0.57 *** (0.4, 0.75)	0.48 *** (0.35, 0.61)	0.59 *** (0.32, 0.86)	0.49 *** (0.27, 0.70)
65–69	0.66 *** (0.51, 0.81)	0.69 *** (0.57, 0.81)	0.72 *** (0.48, 0.95)	0.76 *** (0.55, 0.98)
70–74	0.72 *** (0.59, 0.85)	0.88 *** (0.75, 1.00)	0.84 *** (0.61, 1.06)	1 *** (0.74, 1.26)
75–79	0.72 *** (0.59, 0.84)	0.99 *** (0.84, 1.14)	0.89 *** (0.65, 1.13)	1.18 *** (0.85, 1.50)
80–84	0.69 *** (0.55, 0.83)	1.05 *** (0.86, 1.23)	0.97 *** (0.69, 1.25)	1.32 *** (0.91, 1.73)
85–89	0.71 *** (0.55, 0.86)	1.15 *** (0.92, 1.38)	0.97 *** (0.63, 1.30)	1.41 *** (0.92, 1.91)
90–94	0.72 *** (0.53, 0.90)	1.12 *** (0.85, 1.39)	0.96 *** (0.56, 1.36)	1.37 *** (0.78, 1.96)
**Period**				
1992	−0.22 *** (−0.33, −0.11)	−0.02 (−0.15, 0.1)	−0.19 (−0.39, 0.01)	−0.01 (−0.26, 0.24)
1997	−0.12 *** (−0.19, −0.05)	−0.04 (−0.12, 0.03)	−0.07 (−0.2, 0.05)	−0.01 (−0.16, 0.14)
2002	0.00 (−0.04, 0.03)	0.00 (−0.03, 0.03)	0.06 * (0.01, 0.10)	0.04 (−0.01, 0.09)
2007	0.04 * (0, 0.07)	0.00 (−0.03, 0.03)	0.06 * (0.01, 0.11)	−0.02 (−0.07, 0.04)
2012	0.08 * (0.01, 0.15)	−0.01 (−0.08, 0.07)	0.04 (−0.09, 0.16)	−0.01 (−0.16, 0.14)
2017	0.23 *** (0.12, 0.34)	0.08 (−0.05, 0.20)	0.11 (−0.09, 0.31)	0.01 (−0.25, 0.26)
**Cohort**				
1898–1902	0.48 * (0.06, 0.90)	0.41 (−0.03, 0.85)	0.63 (−0.25, 1.50)	0.54 (−0.39, 1.46)
1903–1907	0.53 ** (0.13, 0.94)	0.53 * (0.12, 0.95)	0.67 (−0.18, 1.52)	0.66 (−0.23, 1.55)
1908–1912	0.54 ** (0.14, 0.93)	0.59 ** (0.19, 0.99)	0.66 (−0.18, 1.49)	0.71 (−0.15, 1.57)
1913–1917	0.53 ** (0.14, 0.91)	0.63 ** (0.24, 1.02)	0.63 (−0.19, 1.46)	0.73 (−0.11, 1.57)
1918–1922	0.53 ** (0.15, 0.91)	0.64 ** (0.26, 1.03)	0.62 (−0.21, 1.45)	0.72 (−0.12, 1.55)
1923–1927	0.5 * (0.12, 0.89)	0.62 ** (0.23, 1.01)	0.57 (−0.26, 1.41)	0.68 (−0.16, 1.52)
1928–1932	0.43 * (0.03, 0.83)	0.57 ** (0.17, 0.96)	0.48 (−0.37, 1.33)	0.62 (−0.24, 1.48)
1933–1937	0.35 (−0.06, 0.76)	0.51* (0.09, 0.92)	0.4 (−0.47, 1.27)	0.57 (−0.32, 1.46)
1938–1942	0.22 (−0.20, 0.65)	0.39 (−0.04, 0.82)	0.29 (−0.62, 1.19)	0.46 (−0.47, 1.39)
1943–1947	0.19 (−0.25, 0.64)	0.35 (−0.11, 0.81)	0.26 (−0.68, 1.20)	0.42 (−0.56, 1.40)
1948–1952	0.23 (−0.24, 0.70)	0.36 (−0.13, 0.85)	0.29 (−0.69, 1.27)	0.43 (−0.60, 1.47)
1953–1957	0.1 (−0.40, 0.59)	0.18 (−0.34, 0.71)	0.12 (−0.90, 1.15)	0.23 (−0.87, 1.33)
1958–1962	−0.11 (−0.64, 0.42)	−0.09 (−0.66, 0.47)	−0.14 (−1.22, 0.94)	−0.12 (−1.29, 1.06)
1963–1967	−0.11 (−0.67, 0.45)	−0.12 (−0.72, 0.49)	−0.12 (−1.25, 1.01)	−0.1 (−1.35, 1.15)
1968–1972	−0.28 (−0.87, 0.32)	−0.34 (−1.01, 0.32)	−0.35 (−1.55, 0.84)	−0.38 (−1.74, 0.97)
1973–1977	−0.52 (−1.18, 0.13)	−0.63 (−1.45, 0.19)	−0.67 (−1.98, 0.63)	−0.75 (−2.37, 0.88)
1978–1982	−0.74 (−1.52, 0.04)	−0.9 (−2.03, 0.22)	−0.84 (−2.36, 0.68)	−1.02 (−3.22, 1.18)
1983–1987	−0.79 (−1.77, 0.19)	−1.02 (−2.48, 0.44)	−0.95 (−2.97, 1.08)	−1.21 (−4.30, 1.89)
1988–1992	−0.88 (−2.76, 1.00)	−1.13 (−3.69, 1.42)	−1.05 (−5.16, 3.06)	−1.37 (−7.16, 4.41)
1993–1997	−1.2 (−9.21, 6.82)	−1.54 (−9.38, 6.30)	−1.5 (−18.46, 15.46)	−1.83 (−18.78, 15.12)
AIC	6.94	4.93	7.04	5.16
BIC	2099.21	77.97	2364.42	160.07
Deviance	2333.19	311.96	2598.40	394.06

Note: * *p* < 0.05, ** *p* < 0.01, *** *p* < 0.001. Coef, coefficient; AIC, Akaike’s information criterion; BIC, Bayesian information criterion.

## Appendix A

**Table A1:** The average annual percent changes (AAPCs) in incidence and mortality from LC in China, 1990–2017.

Age Group	Mortality (AAPC)			Incidence (AAPC)		
	Both Gender	Males	Females	Both Gender	Males	Females
Age-standardized Rate	−0.8 (−0.9, −0.6)	−0.5 (−0.6, −0.3)	−1.3 (−1.6, −1.0)	−0.2 (−0.2, −0.1)	0.2 (0.1, 0.3.0)	−1.1 (−1.2, −1.0)
5–9	−3.6 (−4.3, −2.9)	−3.8 (−4.5, −3.0)	−3.6 (−4.9, −2.4)	−2.8 (−3.2, −2.5)	−2.8 (−3.2, −2.5)	−3.1 (−3.4, −2.8)
10–14	−2.6 (−3.2, −2.0)	−2.9 (−3.5, −2.3)	−2.7 (−4.1, −1.3)	−1.7 (−1.9, −1.4)	−1.9 (−2.2, −1.5)	−1.6 (−1.9, −1.3)
15–19	−2.7 (−3, −2.4)	−2.3 (−2.7, −1.9)	−3.8 (−4.4, −3.3)	−1.6 (−1.8, −1.4)	−1.1 (−1.4, −0.9)	−2.9 (−3.2, −2.6)
20–24	−3.4 (−4.6, −2.2)	−2.9 (−3.5, −2.4)	−4.5 (−6.3, −2.7)	−2.3 (−2.6, −2.0)	−1.8 (−2.1, −1.4)	−3.3 (−3.5, −3.1)
25–29	−2.2 (−4, −0.3)	−1.8 (−3, −0.5)	−3.3 (−4.2, −2.5)	−1.4 (−1.8, −1.0)	−0.9 (−1.3, −0.5)	−2.8 (−3.2, −2.4)
30–34	−2.5 (−3.6, −1.5)	−2.1 (−3.1, −1.1)	−3.7 (−4.5, −3.0)	−1.4 (−1.7, −1.1)	−1.0 (−1.3, −0.7)	−3.0 (−3.2, −2.7)
35–39	−2.7 (−3.1, −2.3)	−2.4 (−2.8, −1.9)	−3.9 (−4.2, −3.6)	−1.7 (−1.9, −1.4)	−1.3 (−1.6, −1.1)	−3.1 (−3.2, −2.9)
40–44	−2.9 (−3.7, −2.2)	−2.6 (−3.5, −1.8)	−3.9 (−4.4, −3.4)	−1.5 (−1.8, −1.3)	−1.1 (−1.3, −0.9)	−2.9 (−3, −2.8)
45–49	−1.6 (−1.9, −1.3)	−1.3 (−1.6, −1.0)	−2.8 (−3.2, −2.3)	−0.8 (−1, −0.5)	−0.3 (−0.5, −0.2)	−2.4 (−2.6, −2.2)
50–54	−0.9 (−1.3, −0.5)	−0.5 (−0.9, −0.1)	−1.9 (−2.8, −1.1)	−0.3 (−0.6, 0.0)	0.3 (0, 0.7)	−1.6 (−1.9, −1.3)
55–59	−1.5 (−2.3, −0.6)	−1.2 (−1.9, −0.4)	−2.7 (−3.3, −2.1)	−0.6 (−0.8, −0.5)	−0.2 (−0.3, 0.0)	−2.0 (−2.1, −1.8)
60–64	−0.4 (−0.6, −0.2)	0 (−0.3, 0.3)	−1.4 (−1.6, −1.2)	0.1 (0, 0.2)	0.6 (0.5, 0.7)	−1.0 (−1.2, −0.9)
65–69	0.0 (−0.4, 0.5)	0.5 (0.2, 0.9)	−0.7 (−1, −0.3)	0.7 (0.4, 0.9)	1.1 (0.9, 1.4)	−0.5 (−0.7, −0.2)
70–74	−0.5 (−0.7, −0.2)	−0.3 (−0.5, −0.1)	−1.0 (−1.3, −0.6)	0 (−0.1, 0.1)	0.3 (0.2, 0.4)	−0.9 (−1.1, −0.7)
75–79	−0.2 (−0.6, 0.1)	0.1 (−0.4, 0.5)	−0.9 (−1.2, −0.6)	0.2 (0.2, 0.3)	0.7 (0.6, 0.8)	−0.8 (−0.8, −0.7)
80–84	0.2 (−0.2, 0.6)	0.4 (0, 0.8)	−0.3 (−0.9, 0.4)	0.9 (0.8, 1.0)	1.2 (1.1, 1.3)	0.3 (0.2, 0.3)
85–89	0.3 (0, 0.7)	0.5 (0.2, 0.8)	−0.1 (−0.5, 0.4)	1.0 (0.9, 1.0)	1.3 (1.3, 1.4)	0.4 (0.4, 0.5)
90–94	0.7 (0.3, 1.1)	1.0 (0.6, 1.4)	0.5 (0.3, 0.7)	1.5 (1.4, 1.6)	1.8 (1.7, 1.9)	1.2 (1.1, 1.3)
